# TNF signaling maintains local restriction of bacterial founder populations in intestinal and systemic sites during oral *Yersinia* infection

**DOI:** 10.1128/mbio.01779-25

**Published:** 2025-09-09

**Authors:** Stefan T. Peterson, Katherine G. Dailey, Karthik Hullahalli, Daniel Sorobetea, Rina Matsuda, Jaydeen Sewell, Winslow Yost, Rosemary O'Neill, Suhas Bobba, Nicolai Apenes, Matthew E. Sherman, George I. Balazs, Charles-Antoine Assenmacher, Arin Cox, Matthew Lanza, Sunny Shin, Matthew K. Waldor, Igor E. Brodsky

**Affiliations:** 1 Department of Pathobiology, School of Veterinary Medicine, University of Pennsylvania 6572, Philadelphia, Pennsylvania, USA; 2 Division of Infectious Diseases, Brigham and Women's Hospital 1861, Boston, USA; 3 Department of Microbiology, Harvard Medical School 1811 , Boston, USA; 4 Department of Microbiology, Perelman School of Medicine, University of Pennsylvania 6572, Philadelphia, Pennsylvania, USA; 5 Howard Hughes Medical Institute 2405, Chevy Chase, Maryland, USA; NYU Langone Health, New York, New York, USA

**Keywords:** *Yersinia*, infectious disease, tumor necrosis factor, gastrointestinal infection, infection dynamics, bacterial infection, innate immunity

## Abstract

Enteroinvasive bacterial pathogens are responsible for an enormous worldwide disease burden that critically affects the young and immunocompromised. *Yersinia pseudotuberculosis* is a gram-negative enteric pathogen closely related to the plague agent *Y. pestis* that colonizes intestinal tissues, induces the formation of pyogranulomas along the intestinal tract, and disseminates to systemic organs following oral infection of experimental rodents. Prior studies proposed that systemic tissues were colonized by a pool of intestinal replicating bacteria distinct from populations within Peyer’s patches and mesenteric lymph nodes. Whether bacteria within intestinal pyogranulomas serve as the source for systemic dissemination and the relationship between bacterial populations within different tissue sites is poorly defined. Moreover, the host factors that regulate *Yersinia* colonization and dissemination are not well understood. Here, we demonstrate using sequence tag-based analysis of microbial populations in R (STAMPR) that remarkably small founder populations independently colonize intestinal and systemic tissues. Notably, intestinal pyogranulomas contain clonal populations of bacteria that are restricted and do not spread to other tissues. However, *Yersinia* populations are shared among systemic organs and the blood, suggesting that systemic dissemination occurs via hematogenous spread. Finally, we demonstrate that TNF signaling is a key contributor to the bottlenecks limiting both initial colonization and subsequent dissemination of orally acquired bacterial populations. Altogether, this study reveals previously undescribed aspects of infection dynamics of enteric bacterial pathogens.

**IMPORTANCE:**

Dissemination of bacteria following intestinal infection can lead to severe disease, including sepsis, organ damage, and death. However, the intestinal bacterial population dynamics governing the colonization of mucosal and systemic tissues and the intestinal sites that seed systemic spread are not clear. *Yersinia pseudotuberculosis* is a rodent and human intestinal pathogen closely related to the plague agent and provides a natural rodent-adapted model to study systemic bacterial dissemination. Our findings define the infection dynamics of enteric *Yersinia* and the impact of the innate immune system on *Yersinia* colonization of the intestine and systemic organs.

## INTRODUCTION

Enteric bacterial pathogens account for an estimated 3.6 million illnesses per year in the United States and are responsible for 64% of both hospitalizations and deaths caused by contaminated food (1); this burden is even greater in developing regions (2). These include gram-negative bacteria like *Yersinia pseudotuberculosis* (*Y. ptb*), a natural pathogen of humans and wild animals (3). In immunocompromised individuals, *Y. ptb* can disseminate from the intestines leading to septicemia, hepatic or splenic abscesses, septic arthritis, or meningitis (4). The route of extraintestinal spread and the host-derived bottlenecks that limit this remain incompletely defined. Because *Y. ptb* is a natural rodent pathogen, experimental rodent models provide a tractable approach to investigate host and bacterial mechanisms that govern in-host population dynamics during infection. However, standard microbiological approaches are not sufficient to infer the population dynamics of pathogen spread, which requires the utilization of more recently developed tools (5).

Inserting unique nucleotide sequences that serve as specific identifying ‘barcodes’ into fitness-neutral sites of the bacterial chromosome enables the introduction of allelic diversity into an otherwise homogenous population (6). This allows us to track the dissemination of unique clones throughout the host following infection using high-throughput sequencing to determine the clones that seed each site of infection (the founding population) (7). The analysis of the location and abundance of individual clones can clarify the dynamics of dissemination.

Successful enteric pathogens must survive the low pH of the stomach and compete with the intestinal microbiota and antimicrobial peptides to enter the intestinal epithelium (8, 9). *Yersinia* oral infection in mice leads to high colonization of Peyer’s patches and mesenteric lymph nodes prior to systemic infection (10–14). Interestingly, the *Y. ptb* founding populations in the liver and spleen are reported to be similar to the ileal portion of the small intestine (10). We recently found that pyogranulomatous lesions (PGs) containing large numbers of replicating *Y. ptb* were enriched in the distal ileum and had not previously been described as a site for *Y. ptb* intestinal colonization and replication (15). Moreover, the pleiotropic inflammatory cytokine tumor necrosis factor (TNF) was required to form functional PGs, control *Y. ptb* burden, and for host survival (16).

To test the potential contribution of PG *Y. ptb* populations to systemic dissemination, we generated a *Y. ptb* barcoded library containing approximately 56,000 unique barcodes and employed the sequence tag-based analysis of microbial populations in R (STAMPR) pipeline (7). We found that a surprisingly small number of bacterial founders independently seed sites of the small intestine and that each pyogranuloma contained microcolonies derived from a single bacterium. Notably, despite containing high numbers of bacteria, both Peyer’s patches and PGs restricted bacterial dissemination, as they did not share bacterial populations with any other tissue sites. Our data indicate that rather than disseminating from initial sites of replication in intestinal tissues, a distinct bacterial population directly enters the blood stream and disperses systemically. Intriguingly, our data further demonstrate that TNF signaling controls bacterial infection by both limiting the size of the founding populations that initially colonize the tissues, and by impeding their systemic dissemination. Altogether, this study uncovers key aspects of intestinal bacterial dissemination following oral infection and demonstrates that despite initially supporting bacterial replication, mucosal intestinal tissue sites are highly restrictive to bacterial dissemination.

## RESULTS

### Bacterial tissue burdens are driven by the extensive replication of small numbers of initial founders

**Fig 1:**
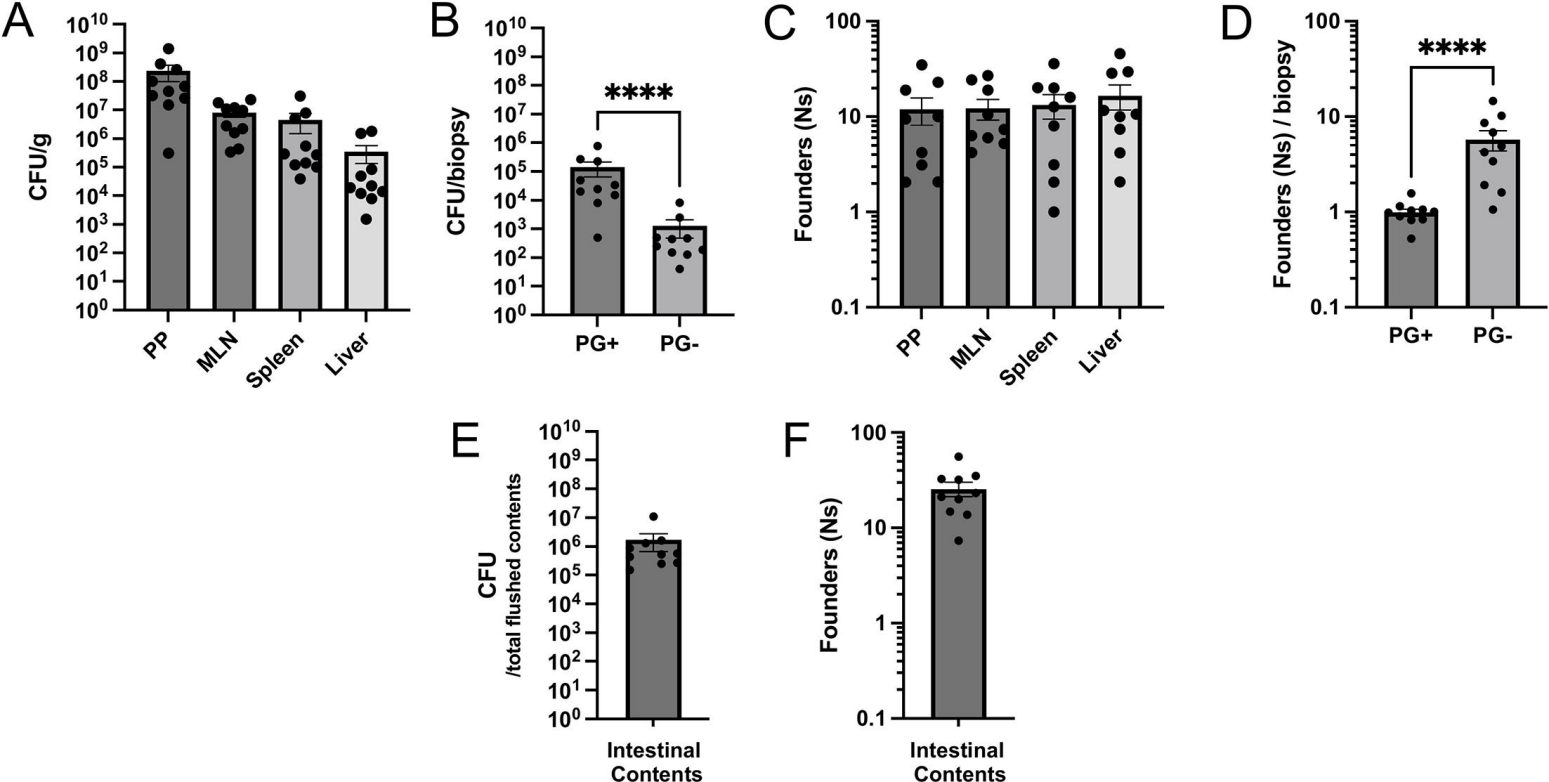
Bacterial tissue burdens are driven by the extensive replication of small numbers of initial founders. (**A**) Bacterial burdens in Peyer’s patch (PP), mesenteric lymph node (MLN), spleen, and liver tissues at day 5 post-infection. (**B**) Bacterial burdens in small intestinal pyogranuloma (PG+) and adjacent non-granulomatous (PG−) tissue isolated day 5 post-infection. Each circle represents the total CFU of 5–10 pooled punch biopsies divided by the number of pooled punch biopsies from one mouse. (**C**) Founding population (Ns) in indicated tissues at day 5 post-infection. (**D**) Founding population (Ns) in small intestinal PG+ and PG− tissue isolated day 5 post-infection. Each circle represents the total Ns of 5–10 pooled punch biopsies divided by the number of pooled punch biopsies from one mouse. (**E**) *Y. ptb* bacterial burden in flushed small intestinal contents (IC) at day 5 post-infection. (**F**) Founding population (Ns) in flushed IC at day 5 post-infection. Samples labeled liver are showing the median lobe as representative of all liver lobes. For all data, circles represent one mouse; bars are mean ± standard error of the mean (SEM); and data are pooled from three experiments. Statistical significance was only calculated in panels B and D using Mann-Whitney tests; ****P < 0.0001.

Previous work suggested non-Peyer’s patch ileal tissue as a potential source of a replicating *Y. ptb* population that spreads systemically (10). To test the possibility that the source for these systemic bacteria might be the ileal PGs (15) and dissect the factors that affect infection dynamics, we generated a library of ~56,000 uniquely barcoded *Y. ptb* using the IP2777 strain (IP2777-STAMPR). We validated the ability of this library to accurately determine founding population (the number of unique bacteria from the inoculum that give rise to the observed population at a site of infection) sizes up to 10^6^ (Fig. S1A), where Ns is the founding population calculated through a computational resampling approach that determines the sampling depth required to observe a given number of barcodes in a particular sample. Mice infected with 2 × 10^8^ CFU of IP2777-STAMPR showed similar burdens in intestinal and systemic tissues compared to the parental IP2777 strain (15) (Fig. 1A), indicating that the barcodes did not affect colonization. Consistent with our previous findings, we observed that intestinal pyogranulomas (PG+) harbor greater than 100-fold higher burden of *Y. ptb* compared to adjacent non-pyogranulomatous tissue (PG−; Fig. 1B).

Tissues from mice infected with IP2777-STAMPR were collected, and the barcodes were sequenced and analyzed using the STAMPR pipeline (7). Bottlenecks were quantified by calculating the founding population (Ns). High Ns values indicate a higher number of founders and, therefore, less restrictive infection bottlenecks than lower Ns values. Notably, the founding populations in both intestinal and systemic sites were very small fractions of the inoculum size: the Peyer’s patches (PP), mesenteric lymph nodes (MLN), spleen, and liver each had approximately 12–17 founders on average per site, with a range of 1–40 in any given mouse (Fig. 1C). This represents a sharp narrowing of the population, with only 0.000008% of the clones in the inoculum being found in the tissues following infection. Intestinal biopsies containing pyogranulomatous lesions (PG+) or non-pyogranulomatous tissue (PG−) were pooled per mouse, and the founding populations at these sites were estimated by dividing the Ns by the number of biopsies from each respective mouse (Fig. 1D). In the PG− intestinal tissue, similar to other tissues, we observed an oligoclonal population with approximately 8–10 unique founders per biopsy. In contrast, PGs primarily contained one founder per biopsy, indicating that bacterial populations within intestinal pyogranulomas are clonal. We considered that a large portion of the inoculum might remain within the intestinal lumen rather than initially colonizing the host tissue. However, the *Y. ptb* burden in the small intestinal contents (IC) during acute infection contained only 10–40 founders (Fig. 1E and F). The low numbers of initial founders were not due to the rapid killing of the inoculum in the GI tract or colonization resistance: we observed high burdens in the stool 1 h and 6 h post-inoculation, indicating that a large number of founders survive and traverse the entire gastrointestinal tract (Fig. S1B and C). Furthermore, broad-spectrum antibiotic pretreatment increased bacterial burdens and the number of founders in the intestinal lumen and in PG− tissues but had a minimal effect on founding populations or burdens at other intestinal and extraintestinal sites, with the exception of the MLN, which had a higher Ns (Fig. S1D through J). Therefore, microbiota influence the bottleneck in the intestinal lumen but not in host tissues. Altogether, these data demonstrate that *Y. ptb* face extremely tight bottlenecks for intestinal colonization and tissue infection and that very small initial founder populations are responsible for all subsequent replication that occurs.

### Peyer’s patches and intestinal pyogranulomas contain distinct bacterial populations

**Fig 2:**
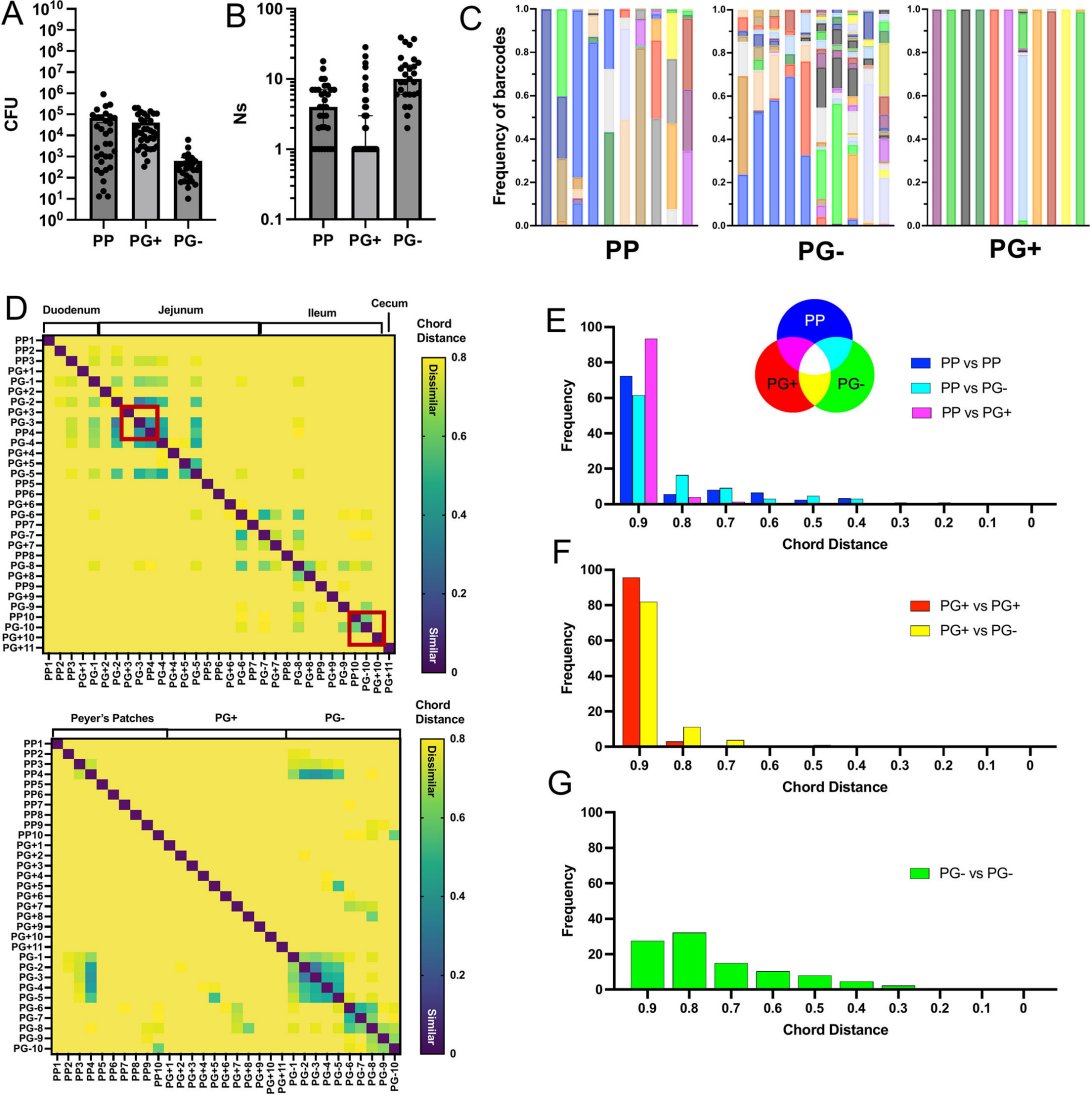
Peyer’s patches and intestinal pyogranulomas contain distinct bacterial populations. (**A**) Bacterial burdens and (**B**) founding population (Ns) in small intestinal PP, PG+, and PG− tissue isolated day 5 post-infection. Each circle represents one biopsy. Bars represent (**A**) mean ± SEM or (**B**) median ± 95% CI. Pooled data from four mice from one experiment. (**C**) Frequency of barcodes per biopsy for mouse 1. Each bar represents one biopsy, and each color represents one barcode. (**D**) Similarity between *Y. ptb* populations in each biopsy, as assessed by the chord distance (CD) for mouse 1, depicted as (top) samples ordered by location along small intestine or (bottom) samples ordered by biopsy type. Red boxes highlight two examples of sharing dynamics where PP shares with adjacent PG− but not with adjacent PG+ (*n* = 4, one experiment, equivalent data of mice 2–4 are depicted in Fig. S2E through J). (**E**) Histograms showing the frequency of binned CD values for each comparison: (**E**) PP vs PP (blue), PP vs PG− (cyan), and PP vs PG+ (magenta); (**F**) PG+ vs PG+ (red) and PG+ vs PG− (yellow); and (**G**) PG− vs PG− (green). For panels E–G, color-coding of comparisons is diagrammed by triple Venn diagram with specific comparisons highlighted in the figure key, bin width = 0.1, and the center of bins is shown on the *x*-axis. Data are cumulative from four mice, one experiment.

*Yersinia* microcolonies in splenic abscesses following intravenous injection of a mixed population of both GFP- and mCherry-expressing bacteria were found to be clonal (11, 17). We observed that the number of unique bacterial founders in pooled intestinal pyogranuloma samples was similar to the total number of biopsies collected, suggesting that bacterial populations within individual pyogranulomas are also clonal. To definitively test whether individual intestinal pyogranulomas contained clonal bacterial populations, we isolated individual PG+, PP, and PG− biopsies from four mice and mapped them along the length of the gastrointestinal tract (Fig. S2A through D). CFU and Ns per biopsy were similar when individual biopsies were harvested (Fig. 2A and B) or when biopsies were pooled and processed together (Fig. 1A through D). Individual PP and PG− typically harbored multiple disparately abundant clones, with 76 and 100% having greater than one barcode, respectively (Fig. 2B and C; Fig. S2E through G). In contrast, individual PG+ primarily contained a single barcode (58%). These data indicate that intestinal pyogranulomas primarily harbor clonal populations of *Y. ptb*.

To test the possibility that bacteria within pyogranulomas might derive from geographically adjacent Peyer’s patches or non-granulomatous tissue, or, conversely, that bacteria within pyogranulomas might seed populations in Peyer’s patches, we compared the bacterial populations along the length of the GI tract using the Cavalli-Sforza chord distance analysis of the STAMPR pipeline (CD) (7). This metric factors in barcode identity and relative abundance when comparing populations. While the highest possible CD between two populations is (2*√2)/∏ ≈ 0.9, a CD of ~0.8 or higher indicates that two populations are meaningfully dissimilar. A lower CD indicates that populations are more similar to one another, with ~0.2 being profoundly similar, and a CD of 0 indicates identical populations. Largely, we observed that adjacent regions of the intestine were more likely to have lower CD, indicating some sharing occurring at these sites, than physically distant biopsies (Fig. 2D; Fig. S2H through J). We did not find a difference in population sharing dynamics based on the intestinal region (duodenum, jejunum, ileum; Fig. S2K), indicating that intestinal geography did not impact the dynamics of bacterial populations.

*Y. ptb* is thought to translocate into intestinal tissue through M cells that predominantly overlay Peyer’s patches (PP) (18), raising the possibility that PP serve as initial sites of intestinal colonization prior to bacterial spread to neighboring intestinal sites. However, PP and other intestinal biopsies had high CD, indicating very little spread to other intestinal tissues from the PP (Fig. 2E). However, we did observe small numbers of PP that had lower CD to other PP and uninflamed intestinal sites (PG−). In contrast, over 93% of PP to PG+ comparisons are completely dissimilar, as indicated by CD values greater than 0.8 (Fig. 2E). For example, in one mouse, we observed shared clones between Peyer’s patches and adjacent uninflamed tissues but no sharing with adjacent pyogranulomas (Fig. 2D, red boxes). Because the *Y. ptb* populations in intestinal pyogranulomas are clonal, while Peyer’s patches are oligoclonal, CD could be masking instances where a founder is present at both sites but represents a small proportion of the clones in the Peyer’s patch. Therefore, we enumerated the cases where a PG founder was also present in a PP within the same mouse. Accordingly, 66% of PGs did not have a shared *Y. ptb* founder with any PP (Fig. S2L). Together, these results indicate that *Y. ptb* populations in Peyer’s patches and intestinal pyogranulomas are largely distinct.

We observed regions of the intestine containing multiple pyogranulomas in proximity (Fig. S2A through D), raising the question of whether geographically related PGs might also be seeded by common founder bacteria. However, CD values among PGs were greater than 0.8 (Fig. 2F), indicating that they were largely dissimilar, and we found only two instances where adjacent pyogranuloma biopsies shared *Y. ptb* founders (Fig. S2H and I). These data indicate that intestinal pyogranulomas are seeded by independent, unique, founder bacteria from the initial inoculum pool. PG+ *Y. ptb* populations were largely distinct from PG− populations (Fig. 2F). However, while pyogranulomas rarely share clones with Peyer’s patches or other pyogranulomas, 60% of PG+ share their top barcode with one or more PG− biopsies (Fig. S2L), indicating either that adjacent PG− tissue could be a source for the bacterial populations within pyogranulomas, or that *Y. ptb* in PGs can escape into non-PG areas of the lamina propria. Interestingly, we observed a wide CD distribution for comparisons between individual PG− biopsies, with 30% of biopsy comparisons less than 0.8 (Fig. 2G). This significantly lower distribution of CDs between PG− biopsies (Fig. S2M and N) indicates that distinct regions of non-inflamed intestinal tissue contain similar *Y. ptb* populations more frequently than either Peyer’s patches or pyogranulomas do. Together, these data show that the *Y. ptb* populations that seed individual Peyer’s patches and PGs are distinct, with unique clonal populations forming in individual pyogranulomas.

### Bacterial populations in the spleen and liver are distinct from those in the intestine

**Fig 3:**
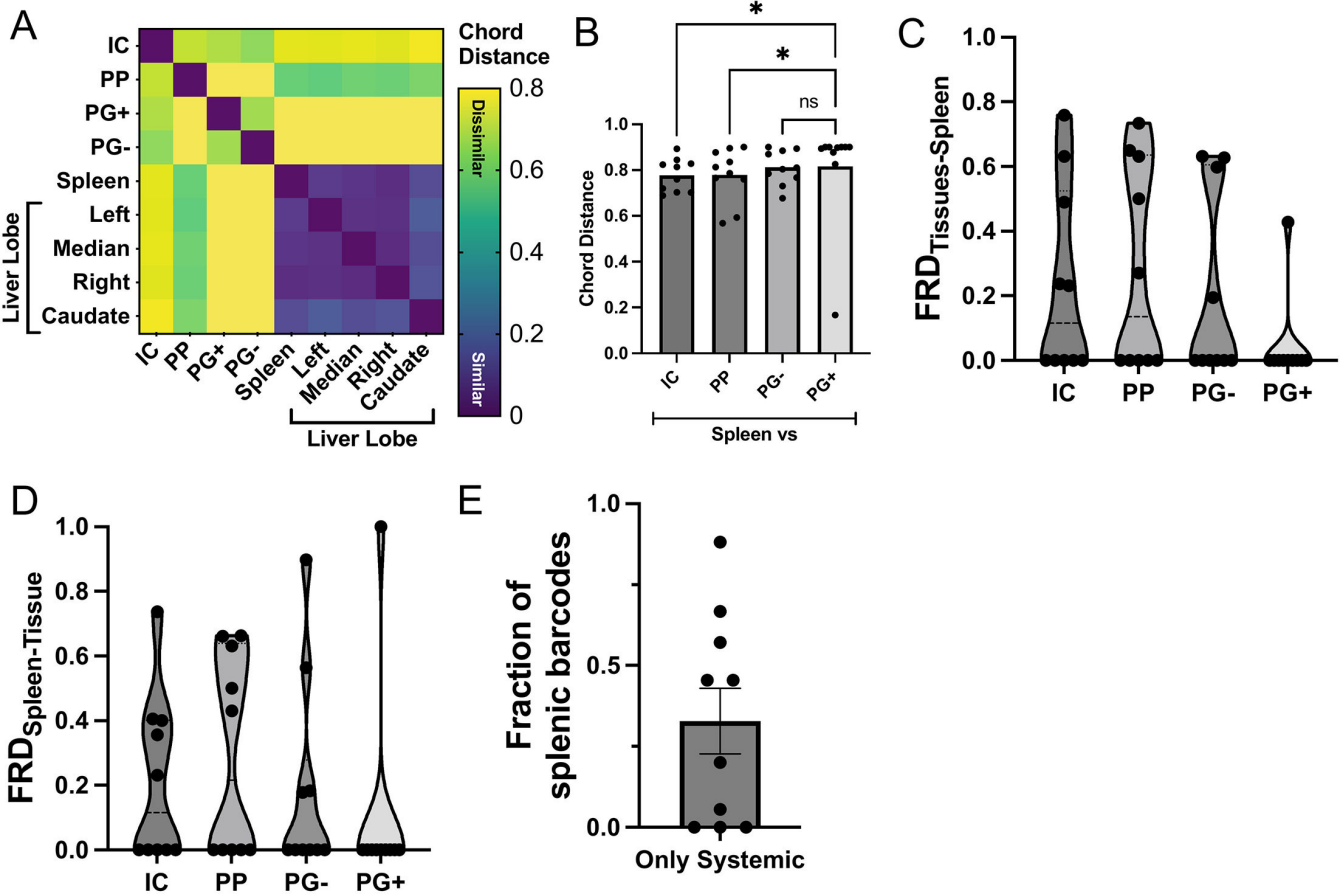
Bacterial populations in the spleen and liver are distinct from those in the intestine. (**A**) Similarity between *Y. ptb* populations in each sample, as assessed by the chord distance (CD) for one representative mouse. (**B**) CD between spleen and indicated samples. (**C and D**) Prevalence of barcodes from (**C**) the spleen in other organs (FRD_organ-spleen_) or (**D**) from various organs in the spleen (FRD_spleen-organ_). (**E**) Fraction of barcodes from the spleen that are not found in other sites outside of the spleen or liver lobes. All data collected at day 5 post-infection; each circle represents one mouse, *n* = 10, pooled data from three independent experiments, where bars are mean ± SEM, and violin plots indicate median ± interquartile range. Statistical significance was calculated using one-way ANOVA with post-hoc Dunn’s multiple comparison test, where ns = not significant and *P < 0.05.

*Y. ptb* is thought to disseminate to systemic sites from an intestinal population that is distinct from PP and MLN^10^, raising the possibility that the systemic sites might be colonized by *Y. ptb* originating from the intestinal pyogranulomas. However, as for other intestinal sites, the CD between *Y. ptb* populations in the spleen and PG+ was at or above 0.8 (Fig. 3A and B), indicating that pyogranulomas are unlikely to be the source of *Y. ptb* that give rise to the splenic population. Furthermore, while PG+ biopsy populations had a chord distance with splenic populations that was significantly higher than that of PP, PG− tissue, or the intestinal contents, all of these locations had a CD with the spleen that was very close to 0.8, indicating that, in general, these tissues did not share populations with the spleen (Fig. 3B). Moreover, the splenic population of *Y. ptb* was not similar to populations in all gastrointestinal organs, including the stomach, cecum, and colon (Fig. S3A), indicating that there is a general lack of similarity between *Y. ptb* populations in gastrointestinal and systemic tissues.

To test the possibility that individual lobes of the liver might harbor distinct subpopulations, as previously observed in the brain in a *Listeria* intravenous infection model (19), we harvested individual liver lobes and assessed their bacterial populations in relation to one another and the spleen. Notably, the spleen and all liver lobes showed very low CD values relative to one another, indicating that the *Y. ptb* populations in each liver lobe and the spleen are derived from similar clones from the initial inoculum (Fig. 3A; Fig. S3B). Additionally, in both spleen and liver, a shared dominant barcode comprised more than 70% of the reads on average (Fig. S3C and D). This level of dominance by a single founder contrasts with the MLN and intestinal contents, where the most abundant barcode was typically less than half of the barcode reads per sample (Fig. S3C and D). These data reflect the differential expansion of a single clone across multiple systemic organs, suggesting a possible priority effect, in which early colonization allows an early founder to have a replicative advantage over subsequent colonizers, and has previously been observed in a *Salmonella* oral infection model (20). Collectively, these data indicate that *Y. ptb* populations are readily exchanged between systemic sites and that the initial colonized intestinal sites that support *Y. ptb* replication do not serve as sources for systemic dissemination.

The CD calculation could potentially mask the relatedness of populations in different tissues that share founders due to the presence of a single high-abundance founder. Therefore, we used the STAMPR pipeline to calculate the log fraction of the relative numbers of clones shared between two sites, termed the fractional resilient distance (FRD) (7). FRD provides insight into the contribution of individual clones to population similarity by quantifying the fraction of barcodes that are shared between two samples. A high FRD_sample1-sample2_ (maximum of 1) indicates that the shared barcodes between samples 1 and 2 represent a large fraction of the total barcodes in sample 2. In this example, FRD_sample2-sample1_ could be low, indicating that these same shared barcodes represent only a small fraction of the total barcodes in sample 1.

Notably, in every mouse, except one, FRD_PG+-Spleen_ is 0, indicating that none of the barcodes in the spleen are shared with intestinal pyogranulomas (Fig. 3C). Likewise, in most mice, the FRD_Spleen-PG+_ is 0, showing that none of the *Y. ptb* clones in the PG+ biopsies are shared with the spleen. Altogether, these findings reveal that intestinal PGs are not a source for systemic bacterial dissemination. Other intestinal sites have FRDs above 0, revealing some level of population overlap with the systemic sites (Fig. 3C and D). However, no one site consistently shared barcodes with the spleen, suggesting that no single mucosal site is a common source for systemic *Y. ptb* dissemination. Unexpectedly, some spleens harbored unique *Y. ptb* founders that were not observed in any other surveyed tissues (Fig. 3E), suggesting either that *Y. ptb* in systemic organs does not need to first replicate in the gastrointestinal tract to disseminate systemically, or that an initial pool from which bacteria might disseminate is no longer present at the time we surveyed the tissue. Together, these data indicate that systemic *Y. ptb* populations do not disseminate from a single consistent mucosal intestinal site, although intestinal contents, Peyer’s patches, and PG− intestinal tissue occasionally shared founders with the spleen. In contrast, the intestinal PGs represent sites of containment that limit the spread of bacteria to other tissues.

### Blood is a conduit for systemic *Y. ptb* populations

**Fig 4:**
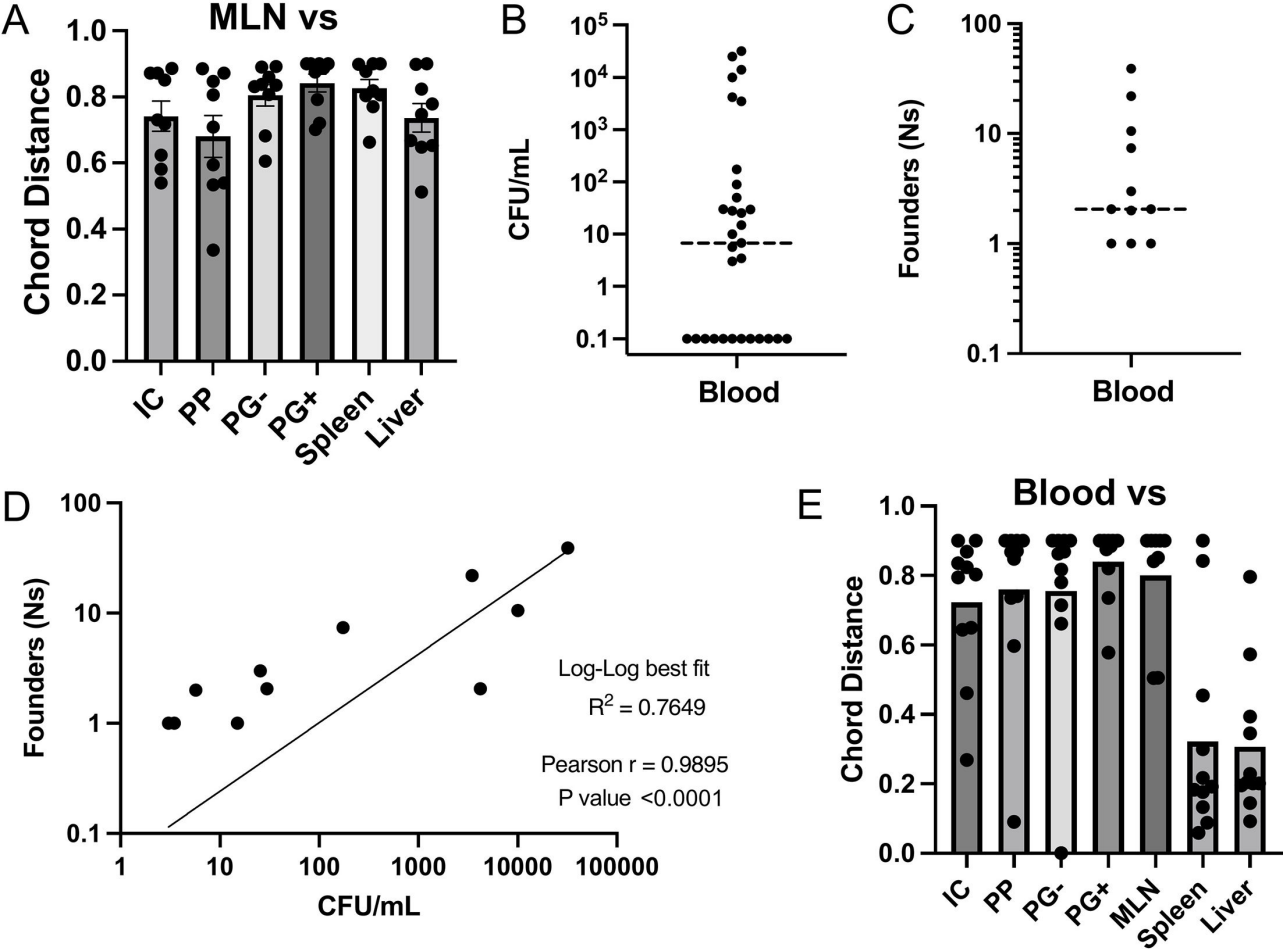
Blood is a conduit for systemic *Y. ptb* populations. (**A**) CD between the MLN and indicated samples. (**B**) Bacterial burdens and (**C**) founding population (Ns) in blood; blood samples with no CFU are represented as 0.1 CFU. (**D**) Log-log regression with Pearson’s correlation of fit between Ns and CFU measured in blood. (**C and D**) Each circle represents one mouse with detectable burden of *Y. ptb* in blood. (**E**) CD between the blood and indicated samples. Samples labeled liver are showing CD comparisons to the median lobe as a representative of all liver lobes. All data were collected at day 5 post-infection from three to four independent experiments (except panel B, which has pooled data from seven experiments), where each circle represents one mouse unless indicated otherwise, and bars are mean ± SEM.

Both the lymphatics and bloodstream have been proposed as potential routes for systemic bacterial dissemination from the intestine (10, 21–23). We sought to utilize the barcoded *Y. ptb* library to distinguish between these possibilities. Consistent with prior findings (10), we found that the MLN had very high CD from all other sites, including mucosal intestinal sites and systemic tissues (Fig. 4A). Collectively, our findings indicate that sites of bacterial infection and replication in the intestinal mucosa are not sources for systemic dissemination and instead limit bacterial dissemination to other tissues. Given the lack of similarity between MLN and systemic sites, we also conclude that the lymphatics themselves are unlikely to serve as a conduit for dissemination.

We therefore considered the possibility that the spread of bacteria from the initial founder pool could occur close to the time of initial inoculation via the bloodstream. Interestingly, nearly 40% of infected animals had no detectable bacteria in the bloodstream, while others contained moderate (10^2^) or high (10^4^) CFU/mL of blood (Fig. 4B). Moreover, the mice that had detectable bacteria in the bloodstream exhibited a range of unique founders, from a single bacterium in several infected mice to 39 unique founders in one mouse, that showed a positive correlation with total CFU in blood (Fig. 4C and D). Notably, a comparison of populations in the blood and spleen or liver revealed very low CD between these sites (Fig. 4E), indicating that bacterial populations are shared between the spleen, liver, and bloodstream, consistent with the possibility that dissemination to systemic sites occurs via the bloodstream.

### TNFR1 contributes to *Y. ptb* infection bottleneck and MLN colonization

While it is well appreciated that the immune system plays a key role in controlling pathogen burden, how specific immune components do so is less defined. The immune system might limit the number of founders in a tissue, limit replication of those founders, prevent dissemination from an initial colonization site, or some combination of these activities. TNF is critical for defense against infection by numerous pathogens, including *Yersinia*, where it limits bacterial tissue burdens and promotes host survival (16, 24–27). Consistently, intestinal pyogranulomas in *Tnfr1*^−/−^ mice have elevated bacterial burden and exhibit an increase in tissue necrosis (16), raising the hypothesis that TNF signaling in pyogranulomas may limit bacterial spread to systemic tissues. Furthermore, TNF signaling is critical for follicular organization in peripheral lymphoid organs (28), including PP and MLN, that may affect control of infection at these sites.

**Fig 5:**
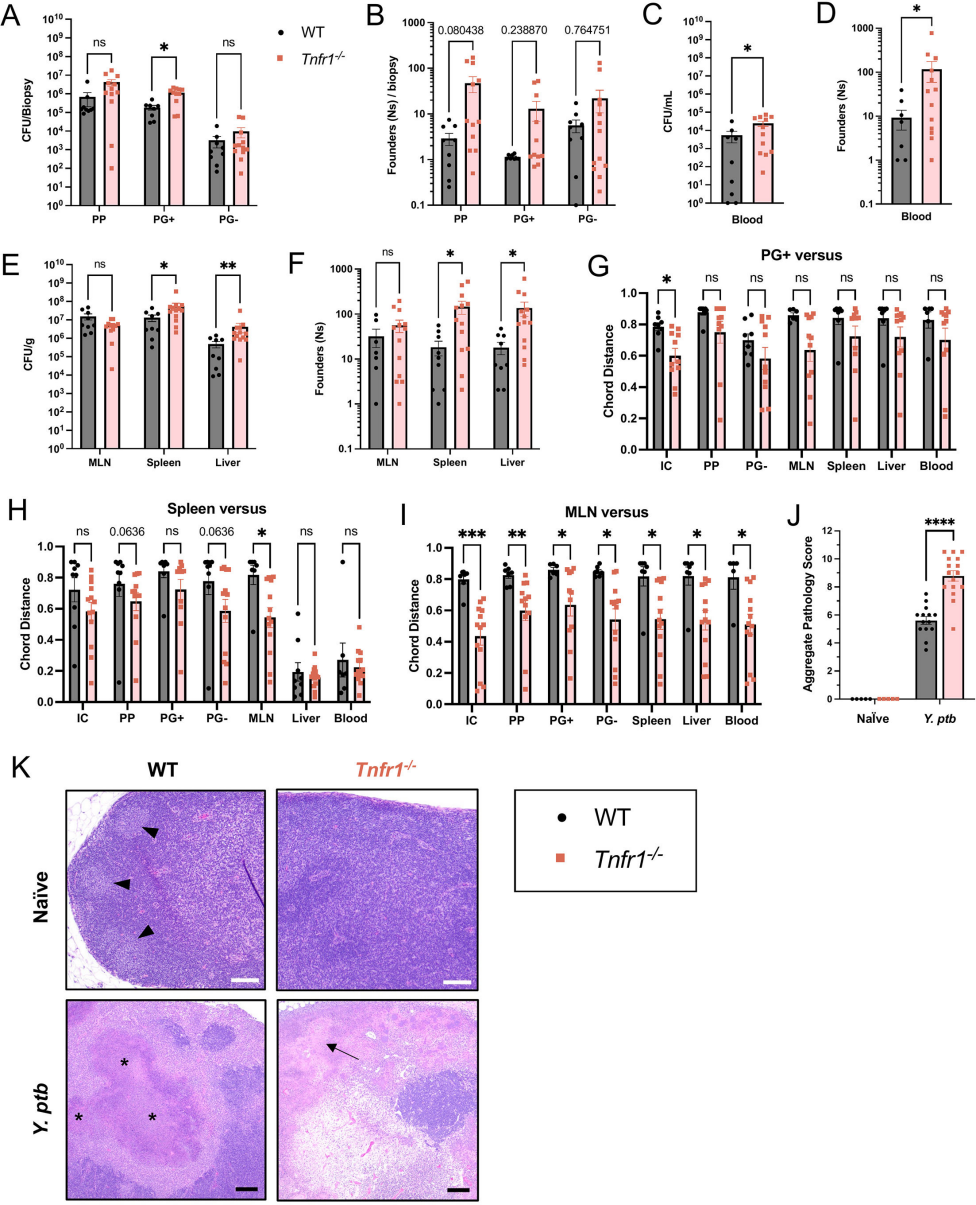
TNFR1 contributes to *Y. ptb* infection bottleneck and MLN colonization. (**A**) Bacterial burdens and (**B**) founding population (Ns) in small intestinal PP, PG+, and PG− biopsies at day 5 post-infection, where each symbol represents the total of 5–10 pooled punch biopsies divided by the number of biopsies from one mouse. (**C**) Bacterial burdens and (**D**) founding population (Ns) in whole blood. (**E**) Bacterial burdens and (**F**) founding populations (Ns) in systemic tissues: mesenteric lymph nodes (MLN), spleen, and liver (median lobe). (**G through I**) Chord distance (CD) between (**G**) PG+, (**H**) spleen, or (**I**) MLN and the indicated systemic (MLN, spleen, median liver lobe, blood) and intestinal (PP and PG−) tissues. All data collected on day 5 post-infection are pooled from three independent experiments. (**J and K**) H&E-stained paraffin-embedded mesenteric lymph node sections from naïve and *Y. ptb*-infected WT and *Tnfr1^−/−^* mice at day 5 after infection were used for (**J**) histopathological scoring and (**K**) imaging. (**J**) Each mouse was scored between 0 and 4 (minimal to extensive) for the metrics shown in Fig. S4E through G. Scores for each mouse were added together to obtain the aggregate score shown. (**K**) Naïve mesenteric lymph nodes from WT mice (left), but not *Tnfr1^−/−^* mice (right), have lymphoid follicles in the cortex (marked by arrowheads). White scale bars = 100 µm. Infected mesenteric lymph nodes (bottom) show pyogranulomatous lesions in WT mice (marked by asterisks) and necrosis in *Tnfr1^−/−^* mice (marked by arrows). Black scale bars = 200 µm. Histological data are from two independent experiments with representative images shown. Each symbol represents one mouse unless otherwise indicated. Bars are mean ± SEM. Statistical significance was calculated using multiple Mann-Whitney tests and ns = not significant, *P < 0.05, **P < 0.01, and ***P < 0.001.

To dissect the impact of TNF signaling on dynamics of bacterial infection as well as the possibility that TNF signaling might be important to contain *Y. ptb* within pyogranulomas, we infected *Tnfr1*^−/−^ mice with STAMP-IP2777 and quantified founding population sizes and dissemination patterns at day 5 post-infection. While the loss of TNF signaling resulted in no significant effect on luminal *Y. ptb* populations in the small intestine (Fig. S4A through C), we observed an increase in the number of founders as well as an increase in CFU in PP and PG+ tissues (Fig. 5A and B). Notably, a large number of *Tnfr1*^−/−^ PGs harbored oligoclonal *Y. ptb* microcolonies more frequently than WT PGs, including several mice with 25–50 individual founders. Similarly, PPs of *Tnfr1*^−/−^ mice harbored a wider range of founders than WT mice, including several mice with over 100 unique individual founders. These data together indicate that TNF signaling limits initial colonization of intestinal sites, although the variance in founder populations in tissues of individual mice limited the statistical significance of this analysis.

Importantly, *Tnfr1*^−/−^ mice also had significantly elevated levels of *Y. ptb* in their blood, with all mice carrying at least some bacterial burden in circulation (Fig. 5C). Moreover, we observed a nearly 10-fold increase in the number of unique founders in the blood of *Tnfr1*^−/−^ mice (Fig. 5D), indicating that TNF signaling limits colonization of the bloodstream. Intriguingly, in the absence of TNFR1 signaling, the liver and the spleen also had higher numbers of unique founders and higher bacterial burdens, indicating that TNF prevents colonization of systemic tissues (Fig. 5E and F). Notably, however, TNF signaling does not affect the degree of bacterial replication, as WT and *Tnfr1*^−/−^ mice had similar numbers of CFU/founder in all tissues, except for *Tnfr1*^−/−^ MLN, which had a significantly lower CFU/founder (Fig. S4D through F). Together, these data indicate that TNFR1 plays a role in limiting initial colonization of tissue sites.

We next examined if the expansion of the colonization bottleneck by the TNF signaling deficiency would result in enhanced dissemination either between intestinal sites or from intestinal sites to systemic tissues. Although there was a trend to lower chord distance (increased similarity) between *Y. ptb* populations in intestinal pyogranulomas and other intestinal tissues in *Tnfr1*^−/−^ relative to WT mice, this reached significance only when comparing pyogranuloma and intestinal luminal contents populations, suggesting that TNFR1 signaling limits the initial colonization of PGs from the intestinal lumen (Fig. 5G). Furthermore, *Tnfr1*^−/−^ intestinal PP and PG *Y. ptb* populations showed a trend of greater similarity to splenic populations, with lower CDs than the same comparison in WT mice (Fig. 5H). Critically, the MLN in *Tnfr1*^−/−^ mice harbored *Y. ptb* populations with significantly elevated sharing with both intestinal and systemic populations, indicating that TNF signaling plays a particularly important role in limiting dissemination between MLN and other tissues (Fig. 5I). Together, these data show that TNFR1 functions to impose infection bottlenecks and limit bacterial dissemination following *Y. ptb* oral infection. Consistent with disrupted intestinal pyogranulomas in the absence of TNF signaling, *Tnfr1*^−/−^ mice exhibited disrupted MLN architecture and increased MLN pathology relative to their wild-type counterparts following *Y. ptb* infection (Fig. 5J and K; Fig. S4G through I).

Together, the distinct influences of TNF signaling on intestinal and systemic sites suggest that TNF signaling functions to restrict invasion by individual founders. Thus, the increase in CFU in the liver and spleen may be attributable to the increase in the number of clones that colonize systemic sites. Notably, the increase in burden in systemic tissues in the absence of TNF signaling corresponds with an increase in sharing of *Y. ptb* founders between the systemic sites and the MLN, highlighting an important role for TNF in limiting systemic bacterial dissemination.

## DISCUSSION

*Y. ptb* is a natural pathogen of both humans and rodents (3) and has been used extensively to dissect molecular mechanisms of bacterial infection and host immune defense (25, 29, 30). Previous studies indicated that systemic bacterial populations derive from a replicating intestinal pool and are distinct from bacterial populations in Peyer’s patches and mesenteric lymph nodes (10). Our recent finding that intestinal pyogranulomas form acutely during *Y. ptb* infection and contain large numbers of bacteria (15) raised the possibility that these pyogranulomas might serve as a source for systemic populations. To test this possibility and further analyze *Y. ptb* infection dynamics, we generated a barcoded population of otherwise isogenic *Y. ptb* and employed the STAMPR analytic pipeline (7).

The vast majority of the initial inoculum survives the acidic environment of the stomach following oral inoculation and rapidly transits through the GI tract. Notably, the tissues are colonized by only 10–20 founders, indicating a ~10^7^-fold narrowing of the population. Each tissue site had its own unique subpopulation of bacteria stochastically colonized by independent founder populations. Surprisingly, while antibiotic depletion of microbiota led to increased *Y. ptb* levels in the intestinal lumen, it did not broadly affect the bacterial tissue burdens, the numbers of unique founders within any tissue site, or spread of bacterial populations between sites, suggesting that the microbiota is not a major bottleneck for tissue colonization by *Y. ptb*.

Consistent with previous observations (10), we found that the MLNs and intestinal sites did not share founder populations with those of systemic sites. Moreover, individual pyogranulomas contained clonal bacterial populations in contrast to PG− intestinal tissue, which had 2–10 unique founders. Intestinal pyogranuloma and Peyer’s patch populations were not similar, suggesting that bacteria from Peyer’s patches do not seed the pyogranuloma or vice versa. However, some members of the bacterial populations in adjacent non-granulomatous intestinal sites were also observed in either Peyer’s patch or pyogranuloma populations, suggesting that there is some limited exchange of bacteria within the intestine. Interestingly, bacteria in the MLNs were not shared with any other tissue, indicating that MLNs are highly restrictive and are a dead end for these bacteria. Notably, both LTβ-deficient mice, which lack Peyer’s patches, and LTα-deficient mice, which lack both Peyer’s patches and MLN, still have systemic *Yersinia* burdens following oral infection (10, 31), suggesting that some *Yersinia* can go directly to systemic sites without replicating in the intestines or MLN.

While we were unable to detect the sharing of bacterial populations between intestinal and systemic sites, the liver and spleen populations had significant similarity with one another and with populations in the bloodstream in the instances in which we could detect these bacteremic populations. In these cases, bacterial burdens positively correlated with the number of unique founders in the blood, suggesting that bloodstream bacterial burden is determined by the entry of unique founders into the circulation. While the mechanism of *Y. ptb* entry into the circulation remains unknown, it may be a result of cell trafficking through interactions with host receptors like CD209 (32) on phagocytes, or through pathogen-specific factors that disrupt host blood vasculature as seen with *Y. pestis* (33). The hematogenous spread of gastrointestinal pathogens has been observed in *Salmonella* (34–36) and *Klebsiella pneumoniae* (37) infection, suggesting a common route of transport to the systemic sites for both intracellular and extracellular pathogens, and that replication at the initial sites of colonization is not required for pathogens to spread to systemic sites.

While *Y. ptb* was often below the level of detection in the systemic circulation of WT mice, *Y. ptb* levels were high in the circulation of TNFR1-deficient mice, corresponding to higher numbers of founders at systemic sites as well as Peyer’s patches and intestinal PGs. Dysregulated PGs in *Tnfr1*^−/−^ mice harbored oligoclonal *Y. ptb* populations, indicating the TNF signaling restricts the colonization of the PGs to a single founder. Nonetheless, these populations were still distinct from other infected tissue sites, indicating that TNF signaling was not required to limit dissemination from PGs to other sites.

Notably, in the absence of TNFR1, the MLN shared significantly more bacterial founders with other tissue sites, indicating that TNF signaling restricts the dissemination of bacteria from the MLN to other tissues. Interestingly, the *Tnfr1*^−/−^ MLN had lower CFUs/founder relative to their WT counterparts likely due to a trend to slightly lower CFU/g and a trend to higher founders in the MLN, though the disordered follicles of the *Tnfr1*^−/−^ MLN could lead to enhanced interactions between *Y. ptb* and phagocytes, potentially limiting bacterial replication (38). Future studies will mechanistically investigate the TNF-dependent restriction of *Y. ptb* lymphatic spread and the potential role of IL-1, which acts downstream of TNF (16).

**Fig 6:**
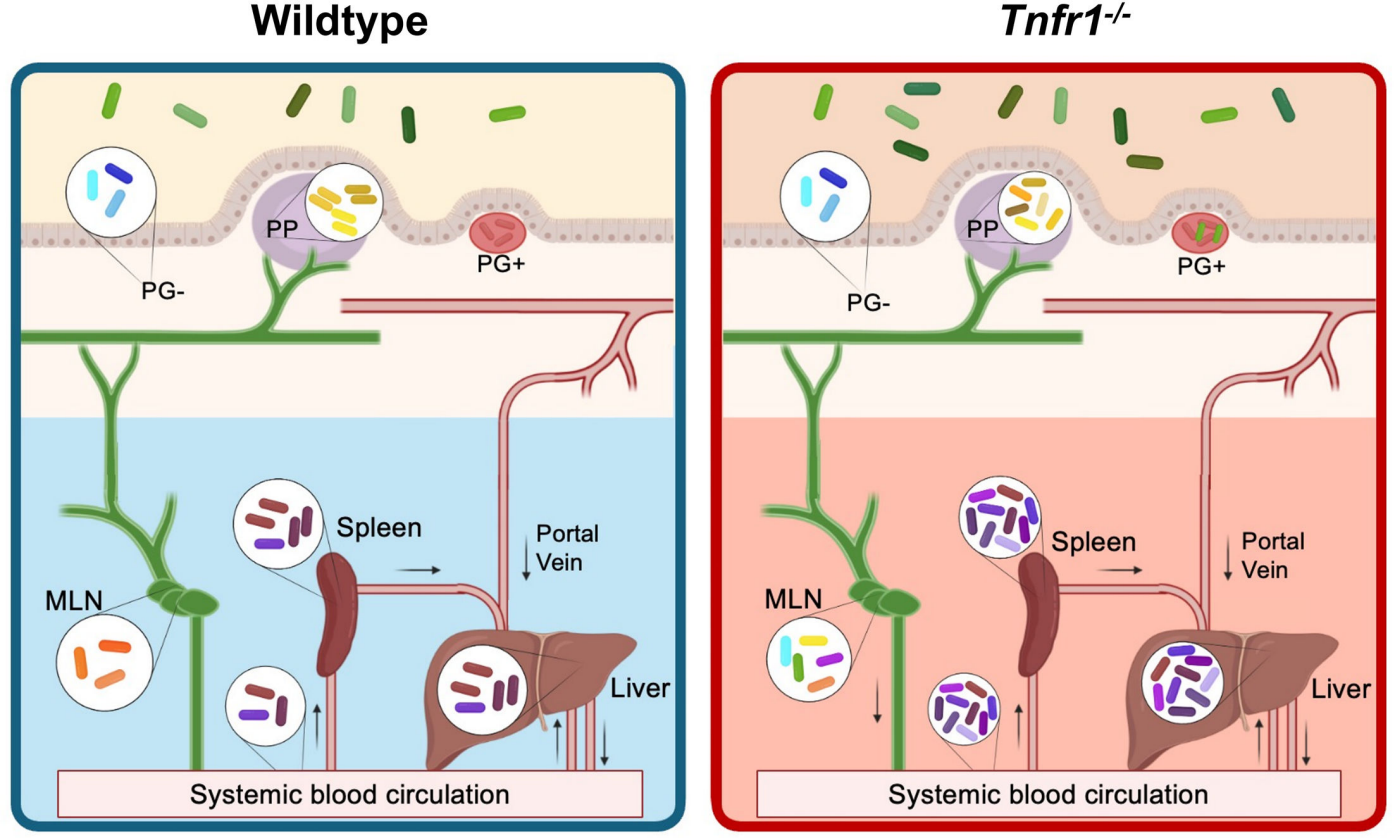
Barcoded *Y. ptb* reveal unique subpopulations across organs, hematogenous spread, and role for TNFR1 in lymphatic restriction of clones. (Left) Wild-type mice are able to control bacterial burdens by maintaining tight bottlenecks to infection. A small number of clones (each depicted by a unique color) are able to seed infection by invading the intestinal tissue and replicating or disseminating directly to systemic organs via the blood. We found that intestinal pyogranulomas (PG+) harbor a clonal population of *Y. ptb* that is contained, thus not the source for systemic dissemination. Meanwhile, mesenteric lymph nodes (MLN) harbor a small population of *Y. ptb* clones that is restricted to this site. (Right) *Tnfr1^−/^*^−^ mice are not able to control bacterial burdens because of a wider bottleneck allowing more clones to colonize sites of infection. Necrotic PG+ biopsies occasionally harbor oligoclonal populations of *Y. ptb. Tnfr1^−/^*^−^ mice have MLN with altered architecture that upon infection harbor founders that are shared with other sites of infection.

Overall, our results support a model where most of the bacteria survive in the stomach to arrive in the intestines shortly after inoculation, but only a select few are able to colonize intestinal tissue and systemic sites (Fig. 6). Each pyogranuloma in the small intestine is seeded by a unique single founder that replicates there. In the adjacent areas of the intestine lacking pyogranulomas, multiple *Y. ptb* clones replicate to a lesser extent. Our findings also suggest that in any given mouse, approximately 10–20 initial founders directly enter systemic circulation to reach the liver and spleen. Our evidence suggests that TNF signaling restricts pathogen translocation to intestinal tissue and systemic sites, particularly from the MLN. Overall, our study reveals key aspects of bacterial population dynamics within the host and highlights the role of specific immune signals in limiting systemic dissemination of gastrointestinal pathogens.

## MATERIALS AND METHODS

### Mice

C57BL/6 (strain #000664) wild type were acquired from the Jackson Laboratory. *Tnfr1^−/−^* mice were previously described (16, 26). All mice were bred at the University of Pennsylvania by homozygous mating and housed separately by genotype. Mice of either sex between 8 and 12 weeks of age were used for all experiments.

### Generation of STAMP library

Wild-type *Y. ptb* (clinical isolate strain 32777, aka IP2777, serogroup O1) (39) has been used extensively in mouse oral infection (15, 16, 40) and provided by Dr. James Bliska (Dartmouth College) (41). A revTet constitutive mCherry construct provided by Dr. Kimberly Davis (Johns Hopkins University) was cloned into a previously described neutral site, YPK_2061 (42), using two-step allelic recombination with a plasmid provided by Dr. Joan Mecsas (Tufts University). This IP2777-mCherry was used to create the barcoded library used throughout this manuscript. The growth of this mutant in 2× YT broth was indistinguishable from the wild-type strain from which it was derived by growth curves. The library “STAMP-IP2777” was created as described previously (19, 43) using the pSM1 plasmid donor library (44). Sequencing indicated that the STAMP-IP2777 library contains ~56,000 unique barcodes (see also Extended methods).

### Mouse infections

STAMP-IP2777 was prepared for oral inoculation by resuspending frozen aliquots in liquid 2× YT broth supplemented with 2 µg/mL triclosan (Millipore Sigma) and 100 µg/mL kanamycin (GoldBio). Bacteria were cultured to stationary phase at 28°C and 250 rpm shaking for 16 h. Bacteria were pelleted and resuspended in phosphate-buffered saline (PBS) at 2 × 10^9^ colony-forming units (CFU) per mL. Mice were fasted for 16 h and subsequently inoculated by oral gavage with 100 µL of inoculum, that is, 2 × 10^8^ CFU per mouse. Following inoculation, the dose was determined by serial dilution and plating.

### Sample harvesting

Samples were harvested as described in Extended methods (Supplemental Material).

All samples had 100 µL serially diluted 10-fold in PBS, plated on LB agar supplemented with 2 µg/mL triclosan and 100 µg/mL kanamycin, and incubated for 2 days at room temperature. Dilutions of each sample were plated in triplicate and expressed as the mean CFU per gram or per biopsy. The remaining 900 µL of homogenized samples was plated on 15 cm dishes with LB agar supplemented with 2 µg/mL triclosan (irgasan) and 100 µg/mL kanamycin and incubated for 2 days at room temperature.

### STAMP sample processing

*Y. ptb* colonies were washed off plates, collected in PBS with 25% glycerol, diluted in water, and boiled for 15 min at 95°C. The barcode-containing region was amplified from the genome using custom forward and reverse primers (35, 37, 43). A sequence tag-based analysis of microbial populations (STAMP) was performed as previously described (37).

### Statistics

Statistical analyses were performed using Prism v9.0 (GraphPad Software). Independent groups were compared by Mann-Whitney *U* test or Kruskal-Wallis test with Dunn’s multiple comparisons test. Statistical significance is denoted as * (*P* < 0.05), ** (*P* < 0.01), *** (*P* < 0.001), **** (*P* < 0.0001), or ns (not significant).

## Data Availability

Sequencing data and original script have been deposited on Dryad (DOI: 10.5061/dryad.2jm63xt21). Any additional information required to reanalyze the data reported in this paper is available from the corresponding author upon request.

## SUPPLEMENTAL MATERIAL

Supplemental Material mbio.01779-25-s0001.pdf Extended methods and supplemental figures.

ASM does not own the copyrights to Supplemental Material that may be linked to, or accessed through, an article. The authors have granted ASM a non-exclusive, world-wide license to publish the Supplemental Material files. Please contact the corresponding author directly for reuse.
