# Supplementary material for: TNF signaling maintains local restriction of bacterial founder populations in intestinal and systemic sites during oral Yersinia infection
Source: mBio. 2025 Sep 9;16(10):e01779-25. doi: 10.1128/mbio.01779-25 (PMC12506003; doi:10.1128/mbio.01779-25)
Supplement: Supplemental Material — Extended methods and supplemental figures. [file mbio.01779-25-s0001.pdf]

Supplemental Materials

TNF signaling maintains local restriction of bacterial founder populations in intestinal and systemic sites during oral *Yersinia* infection

Stefan T. Peterson[a], Katherine G. Dailey[b,c]†, Karthik Hullahalli[b,c]†, Daniel Sorobetea[a]*, Rina Matsuda[a]‡, Jaydeen Sewell[d], Winslow Yost[a], Rosemary O'Neill[a], Suhas Bobba[a,d], Nicolai Apenes[a], Matthew E. Sherman[a,d], George I. Balazs[b,c], Charles-Antoine Assenmacher[a], Arin Cox[a], Matthew Lanza[a]§, Sunny Shin[d], Matthew K. Waldor[b,c,e], Igor E. Brodsky[a]#

[a]Department of Pathobiology, School of Veterinary Medicine, University of Pennsylvania, Philadelphia, PA, USA

[b]Division of Infectious Diseases, Brigham & Women's Hospital, Boston, USA.

[c]Department of Microbiology, Harvard Medical School, Boston, USA.

[d]Department of Microbiology, Perelman School of Medicine, University of Pennsylvania, Philadelphia, PA, USA

[e]Howard Hughes Medical Institute, Chevy Chase, Maryland, USA.

#Address correspondence to Igor E. Brodsky, ibrodsky@vet.upenn.edu

† equal contribution

This file includes:

Extended Methods

Supplemental Figures

Extended Methods

Generation of STAMP library

The pSM1 donor library is composed of ~70,000 unique plasmids transformed into the donor strain MFDλpir. Each pSM1 plasmid carries a site-specific Tn7 transposon containing a random ~25 nucleotide barcode adjacent to a kanamycin resistance cassette. The Tn7 transposon system integrates at a neutral site in the genome downstream of the gene *glmS*[1]. Conjugation was used to introduce the pSM1 library into IP2777-mCherry, and transconjugants containing the transposon were selected using triclosan and kanamycin. Transconjugant colonies were pooled in PBS with 25% glycerol and frozen at −80 °C in aliquots to create the library STAMP-IP2777.

Sample harvesting

Blood was harvested by cardiac puncture upon euthanasia and collected in 250 U/ml Heparin solution (Millipore Sigma). Small intestines were excised, and luminal contents were flushed with sterile PBS into a 50 mL conical for collection. Intestinal contents were then spun down at 3000 relative centrifugal force (RCF) at 4°C for 10 minutes to pellet bacteria and debris. Supernatant was discarded, and the pellet was resuspended in 1 mL of cold, sterile PBS. Resuspended pellets were then homogenized for 40 seconds with 6.35 mm ceramic spheres (MP Biomedical) using a FastPrep-24 bead beater (MP Biomedical). After flushing, small intestines were opened longitudinally along the mesenteric side and placed luminal side down on cutting boards (Epicurean). Small intestinal tissue containing macroscopically visible pyogranulomas (PG+), adjacent non-granulomatous areas (PG−), and Peyer's Patches (PP) were excised using a 2 mm-ø

dermal punch-biopsy tool (Keyes). Biopsies within each mouse were pooled groupwise, unless otherwise stated. Tissues were collected in 1 mL of sterile PBS, weighed, and homogenized for 40 seconds, as previously described[2,3].

Antibiotics treatment

Antibiotics-treated mice were given filter-sterilized water *ad libitum* containing 0.5 g/L Vancomycin (Sigma-Aldrich), 0.5 g/L Neomycin (Sigma-Aldrich), 0.5 g/L Ampicillin (Sigma-Aldrich), 0.25 g/L Metronidazole ((Sigma-Aldrich) for 7 days. Antibiotics were removed from the drinking water for 24 hours prior to inoculation with STAMP-IP2777.

Histology

Tissues were fixed in 10% neutral-buffered formalin (Thermo Fisher Scientific) and stored at 4°C until further processed. Tissue pieces were embedded in paraffin, sectioned and stained with hematoxylin and eosin (H&E) for subsequent assessment of lymph node architecture and histopathological disease scoring by blinded board-certified pathologists. Tissue sections were given a score from 0 to 4 (healthy to severe) for multiple parameters, including degree of inflammation and degree of free bacterial colonies. Healthy mice were characterized by having none or low levels of the parameters described, whereas severely afflicted mice presented with high amounts of the respective parameters.

References

1 Choi, K. H. *et al.* A Tn7-based broad-range bacterial cloning and expression system. *Nat Methods* **2**, 443-448 (2005). https://doi.org/10.1038/nmeth765

2 Sorobetea, D. *et al.* Inflammatory monocytes promote granuloma control of Yersinia infection. *Nat Microbiol* **8**, 666-678 (2023). https://doi.org/10.1038/s41564-023-01338-6

3 Matsuda, R. *et al.* A TNF-IL-1 circuit controls Yersinia within intestinal pyogranulomas. *J Exp Med* **221** (2024). https://doi.org/10.1084/jem.20230679

Supplemental Figure Legends

Figure S1. Validation of the *Y. ptb* barcode library and impact of antibiotic pre-treatment on barcoded *Y.ptb* dissemination. (A) The ability to determine the size of the founding population from STAMP libraries was validated in culture by comparing the number of plated colonies (CFU; known number of founders) to the size of the founding population following STAMPR analysis of those plated populations (Ns) for 3 cultures across 9 doses. (B) Bacterial burdens and (C) founding population (Ns) in stool isolated 1-, 6-, or 12-hours post-infection. Pooled data from four mice from one experiment. (D) Graphical representation of the experimental design for antibiotic (ABX) pretreatment: C57BL/6J mice were given either a cocktail of ABX or control sterile lab drinking water ad libitum for 7 days and returned to normal drinking water one day prior to infection. At day 0, mice were inoculated with $2x10^8$ CFU *Y. ptb* library via oral gavage. Mice were euthanized 5 days following the inoculation and *Y. ptb* populations were assessed. (E) Bacterial burden and (F) founding population (Ns) in flushed small intestinal contents. (G) Bacterial burdens and (H) founding population (Ns) in small intestinal PP, PG+, and PG- biopsies. Each circle represents the total CFU of 5-10 pooled punch biopsies divided by number of pooled punch biopsies from one mouse. (I) Bacterial burden and (J) founding population (Ns) in MLN, spleen, and liver. Unless otherwise indicated, each circle represents one mouse, and all graphs are pooled data from three independent experiments. For bar graphs, bars represent mean ± SEM, and statistical significance was determined using Mann-Whitney tests: ns = not significant, * $p<0.05$, ** $p<0.01$, **** $p<0.0001$.

Figure S2. Intestinal biopsy maps, analysis of barcodes in mouse intestine, and pooled comparisons of chord distance. (A-D) Map showing a representative layout of individually harvested and processed tissue biopsies from the small intestine, cecum, and colon of mice 1-4 on day 5 post-infection. (E-G) Frequency of barcodes per biopsy for mice 2-4 (mouse 1 is depicted in Fig. 1C), where each bar represents one biopsy, and each color represents one barcode. (H-J) Similarity between *Y. ptb* populations in each biopsy as assessed by chord distance (CD) for mice 2-4 (mouse 1 is depicted in Fig. 1D), where (LEFT) data is organized by biopsy type and (RIGHT) data is ordered by location along gastrointestinal tract. (K) CD between biopsies within the indicated small intestinal region. Each circle represents one comparison between biopsies within the indicated region for that mouse. (L) Proportion of PG+ biopsies that share or do not share with PP or PG- biopsies in the same mouse. Sharing was determined by presence of single PG+ founder in other biopsies or, for PG+ containing more than one founder, the most abundant barcode was used to determine sharing. (M-N) CD between the indicated biopsy types. Each circle represents one comparison between the (M) same or (N) different biopsy types as indicated, where biopsies are matched within mouse. Unless otherwise indicated, for all data bars represent mean ± SEM, (pooled for n=4 mice, one experiment collected at 5 days post-infection). Statistical significance was determined using one-way ANOVA with post-hoc Dunn's Multiple Comparison Test, where ns = not significant and **** $p<0.0001$.

Figure S3. Populations of *Y. ptb* in spleen and liver are similar to each other with one highly abundant clone. (A) CD between the spleen and gastrointestinal tract organ tissues. (B) CD between the spleen and individual liver lobes. (C) Frequency of barcodes per tissue for one representative mouse. Each bar represents one sample, and each color represents one barcode. (D) Frequency of most abundant barcode per sample. All samples collected at 5 days post-infection. Data are pooled from three to five experiments, each circle represents one mouse, bars are mean ± SEM unless otherwise indicated.

Figure S4. TNFR1 signaling affects bacterial colony containment in mesenteric lymph nodes but not clone expansion or lymphadenitis. (A) Bacterial burdens and (B) founding populations (Ns) in flushed small intestinal contents at day 5 post-infection. (C-F) Bacterial burden per founder, as determined by CFU/Ns, in (C) flushed small intestinal contents, (D) small intestinal PP, PG+, and PG- biopsies, (E) whole blood, and (F) indicated systemic tissues at day 5 post-infection. Liver samples show data from the median lobe as representative of all liver lobes. Pooled data from three independent experiments. (G-I) H&E-stained paraffin-embedded mesenteric lymph node sections from naïve and *Y.ptb*-infected WT and *Tnfr1*$^{-/-}$ mice at day 5 after infection were scored between 0 and 4 (minimal to extensive) for the metrics shown. Histological data are from two independent experiments. Each symbol represents one mouse, and bars are mean ± SEM. Statistical significance was determined using multiple Mann-Whitney tests and ns = not significant, * $p<0.05$, ** $p<0.01$.

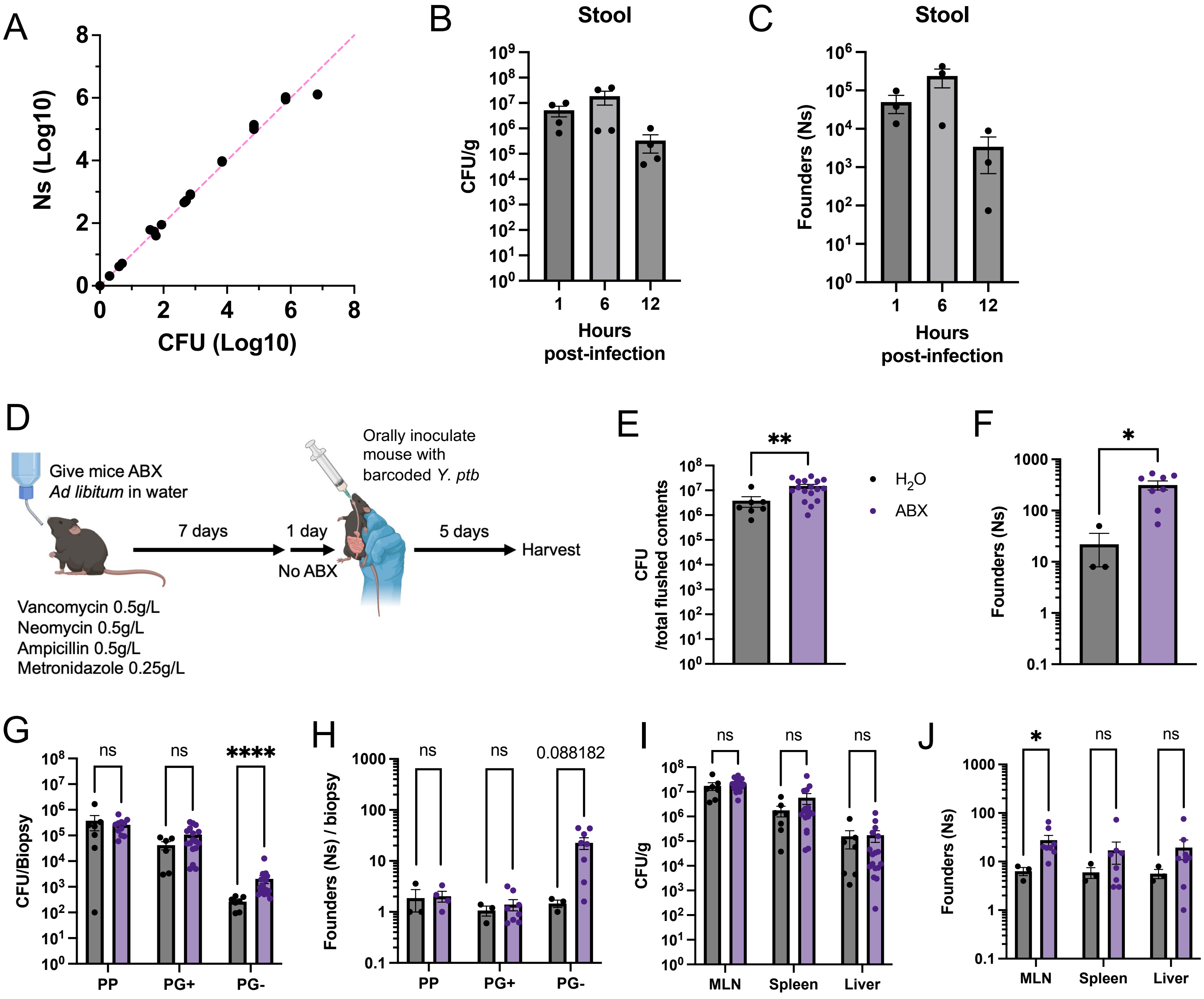
A
Ns (Log10)
CFU (Log10)
B
Stool
CFU/g
Hours
post-infection
C
Stool
Founders (Ns)
Hours
post-infection
D
Give mice ABX
Ad libitum in water
7 days
1 day
No ABX
Orally inoculate
mouse with
barcoded Y. ptb
5 days
Harvest
Vancomycin 0.5g/L
Neomycin 0.5g/L
Ampicillin 0.5g/L
Metronidazole 0.25g/L
E
CFU
/total flushed contents
H2O
ABX
F
Founders (Ns)
G
CFU/Biopsy
PP
PG+
PG-
H
Founders (Ns) / biopsy
0.088182
I
CFU/g
MLN
Spleen
Liver
J
Founders (Ns)

Figure S1

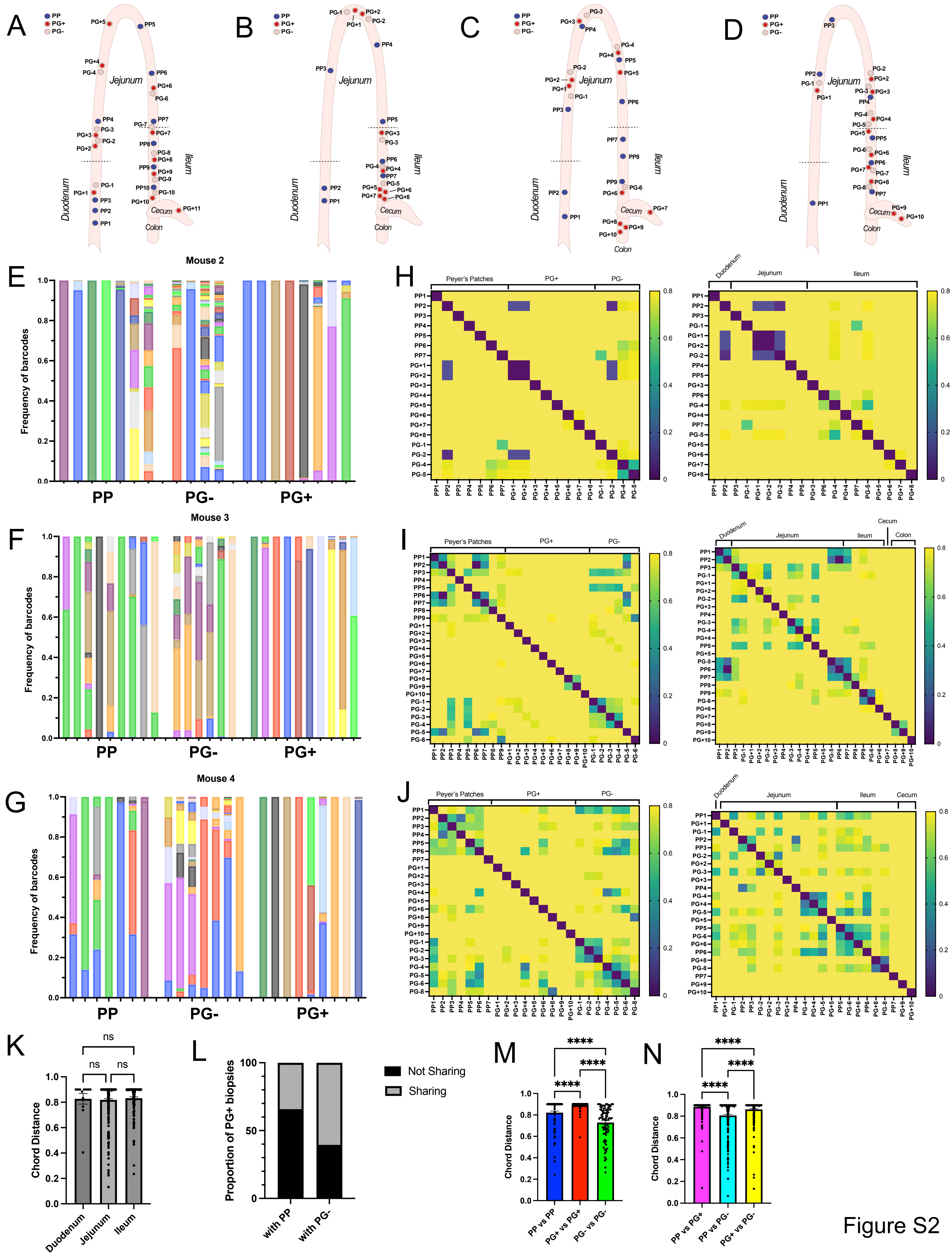

A
B
C
D
Jejunum
Ileum
Duodenum
Cecum
Colon
PP
PG+
PG-
E
F
G
Mouse 2
Mouse 3
Mouse 4
Frequency of barcodes
H
I
J
Peyer's Patches
K
L
M
N
Chord Distance
Proportion of PG+ biopsies
Not Sharing
Sharing
with PP
with PG-
PP vs PP
PG+ vs PG+
PG- vs PG-
PP vs PG+
PP vs PG-
PG+ vs PG-
ns
****

Figure S2

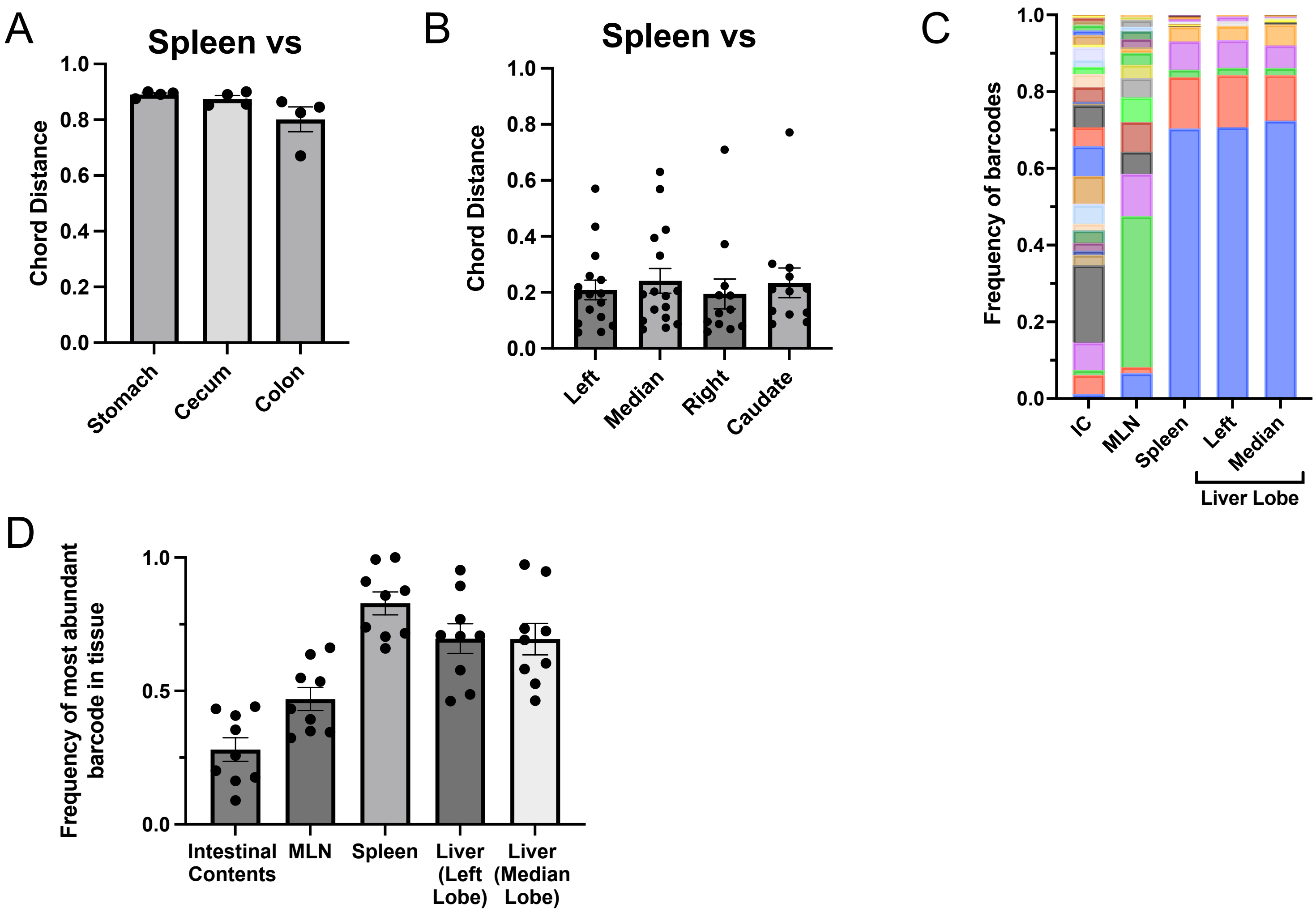

A
Spleen vs
Chord Distance
1.0
0.8
0.6
0.4
0.2
0.0
Stomach
Cecum
Colon
B
Spleen vs
Chord Distance
1.0
0.8
0.6
0.4
0.2
0.0
Left
Median
Right
Caudate
C
Frequency of barcodes
1.0
0.8
0.6
0.4
0.2
0.0
IC
MLN
Spleen
Left
Median
Liver Lobe
D
Frequency of most abundant barcode in tissue
1.0
0.5
0.0
Intestinal Contents
MLN
Spleen
Liver (Left Lobe)
Liver (Median Lobe)

Figure S3

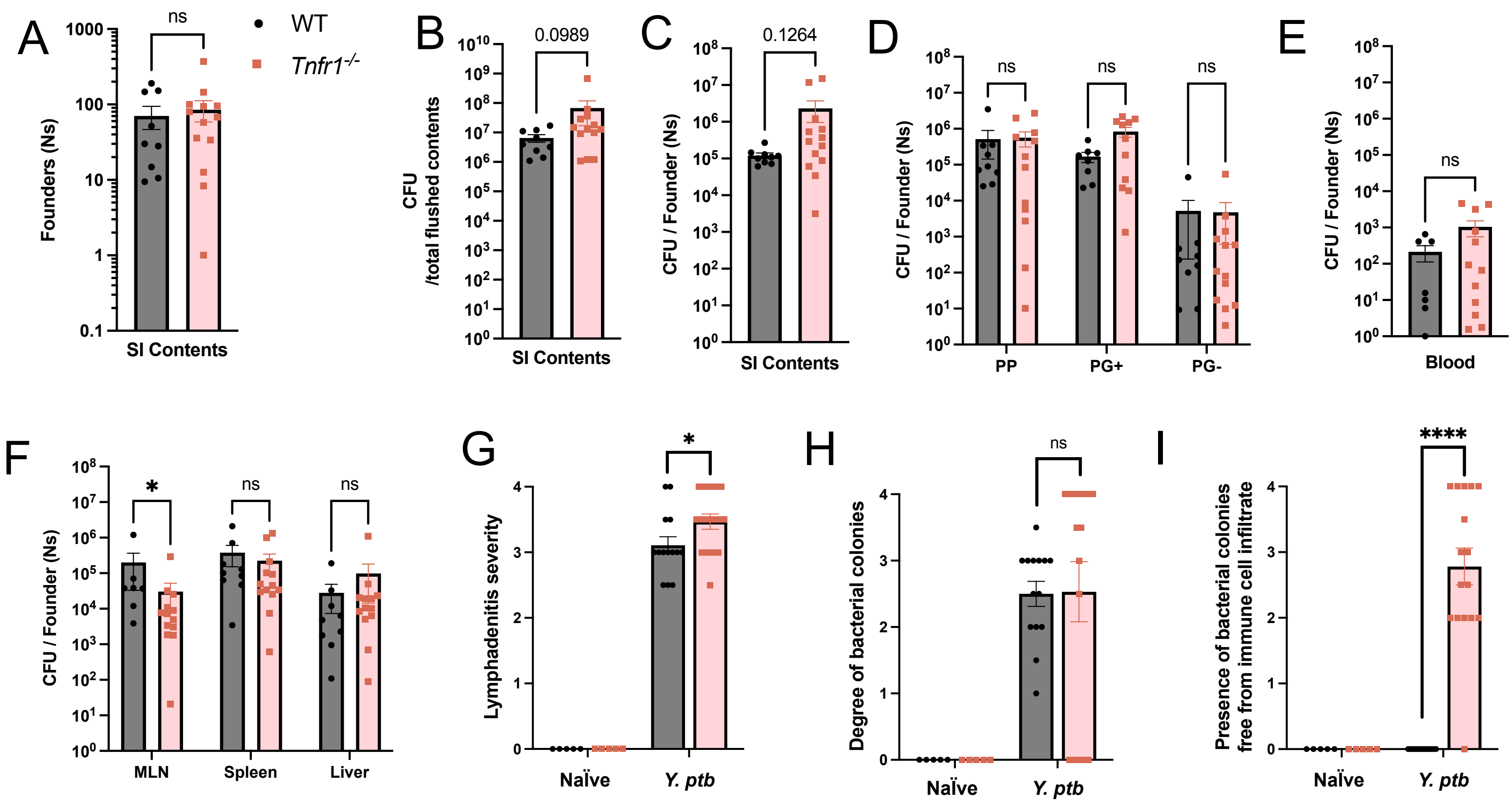
A
B
C
D
E
F
G
H
I
WT
Tnfr1-/-
ns
0.0989
0.1264
****
*
Founders (Ns)
CFU /total flushed contents
CFU / Founder (Ns)
Lymphadenitis severity
Degree of bacterial colonies
Presence of bacterial colonies free from immune cell infiltrate
SI Contents
PP
PG+
PG-
Blood
MLN
Spleen
Liver
Naïve
Y. ptb

Figure S4
